# pCramoll and rCramoll as New Preventive Agents against the Oxidative Dysfunction Induced by Hydrogen Peroxide

**DOI:** 10.1155/2015/520872

**Published:** 2015-10-20

**Authors:** Luís Cláudio Nascimento da Silva, Neyla Maria Pereira Alves, Maria Carolina Accioly Brelaz de Castro, Taciana Mirely Maciel Higino, Cássia Regina Albuquerque da Cunha, Valéria Rêgo Alves Pereira, Nathalia Varejão Nogueira da Paz, Luana Cassandra Breitenbach Barroso Coelho, Maria Tereza dos Santos Correia, Regina Celia Bressan Queiroz de Figueiredo

**Affiliations:** ^1^Laboratório de Bioquímica de Proteínas, Departamento de Bioquímica, Centro de Ciências Biológicas, Universidade Federal de Pernambuco, Avenida Professor Moraes Rêgo, 1235 Cidade Universitária, 50670-901 Recife, PE, Brazil; ^2^Functional Genomics, Department of Biology, Faculty of Science, University of Copenhagen, Ole Maaløes Vej 5, 2200 Copenhagen, Denmark; ^3^Laboratório de Biologia Celular, Departamento de Microbiologia, Centro de Pesquisas Aggeu Magalhães, Fundação Oswaldo Cruz, Pernambuco, Avenida Professor Moraes Rêgo, s/n, Cidade Universitária, 50670-420 Recife, PE, Brazil; ^4^Laboratório de Imunogenética, Departamento de Imunologia, Centro de Pesquisas Aggeu Magalhães, Fundação Oswaldo Cruz, Pernambuco, Avenida Professor Moraes Rêgo, s/n, Cidade Universitária, 50670-420 Recife, PE, Brazil; ^5^Laboratório de Parasitologia, Centro Acadêmico de Vitória, Universidade Federal de Pernambuco, Rua Alto do Reservatório, s/n, Bela Vista, 55608-680 Vitória de Santo Antão, PE, Brazil; ^6^Laboratório de Agregação de Proteínas e Amiloidoses, Instituto de Bioquímica Médica, Universidade Federal do Rio de Janeiro, Cidade Universitária, Ilha do Fundão, 21.941-590 Rio de Janeiro, RJ, Brazil

## Abstract

Oxidative stress plays an important role in the induction of cell death and is associated with various pathologic disorders; therefore, the search for natural products that attenuate the effects produced by oxidant agents is greatly increased. Here, the protective effects of native lectin from *Cratylia mollis* seeds (pCramoll) and recombinant Cramoll 1 (rCramoll) against H_2_O_2_-induced oxidative stress in Vero cells were evaluated. Both lectins significantly attenuated the H_2_O_2_-induced cytotoxicity in a concentration-dependent way. The maximum protective effects were 96.85 ± 15.59% (rCramoll) and 59.48 ± 23.44% (pCramoll). The Live/Dead analysis showed a reduction in the percentage of dead cells from 65.04 ± 3.29% (H_2_O_2_) to 39.77 ± 2.93% (pCramoll) and 13.90 ± 9.01% (rCramoll). The deleterious effects of H_2_O_2_ on cell proliferation were reduced to 10.83% (pCramoll) and 24.17% (rCramoll). Lectins treatment attenuated the excessive superoxide production, the collapse of the mitochondrial membrane potential, and the lysosomal and DNA damage in H_2_O_2_-treated cells. In conclusion, our results suggest that pCramoll and rCramoll blocked H_2_O_2_-induced cytotoxicity through decreasing reactive oxygen species, restoring the mitochondrial potential, preventing the lysosomal damage and DNA fragmentation, and thus promoting cell survival and proliferation.

## 1. Introduction

Oxidative stress is characterized by an imbalance in the redox status of the cell and has been implicated in a range of age-associated and neurodegenerative diseases, such as aging, cancer, diabetes, Alzheimer's disease, and Parkinson's disease [1]. The reactive oxygen species (ROS) are oxygen-containing molecules that are constitutively produced in cells as a result of normal metabolic processes. They include superoxide anions (O_2_^−^), hydroxyl radicals (OH^•^) and hydrogen peroxide (H_2_O_2_; nonradical derivative of oxygen). ROS are known to be responsible for cell toxicity when the generation of ROS exceeds the clearance capacity of the cellular antioxidant systems [2]. H_2_O_2_ is thought to be the major precursor of highly reactive free radicals, such as hydroxyl radicals via Fenton's reaction [3]. ROS may damage relevant classes of biological macromolecules in the cells through direct oxidation of lipids, proteins, and nucleic acids, thereby disrupting cellular function and integrity, which leads to cell death [1, 3]. Nowadays, the search for natural products that attenuate the effects produced by oxidant agents is greatly increased [4, 5].

Lectins are a heterogeneous group of nonimmune proteins and glycoproteins that specifically and reversibly bind with high affinity to carbohydrates without altering the covalent structure of any of their recognized ligands. Lectins can agglutinate cells through binding to cell surface glycoconjugates. They are distributed in plants, animals, and microorganisms [6, 7].

*Cratylia mollis* Mart (Fabaceae family) is a native leguminous forage from the semiarid region of the Northeast of Brazil (Caatinga biome), popularly known as Camaratu bean. Four multiple molecular forms of lectin have been purified from this plant: Cramoll-1, Cramoll-2, Cramoll-3, Cramoll-4; which exhibit different carbohydrate specificities. The isoforms 1, 2, and 4 are nonglycosylated and glucose/mannose specific proteins; and Cramoll 3 is a galactose specific glycoprotein [8, 9]. Cramoll 1,4 (preparation containing isolectins 1 and 4; pCramoll) is isolated in a similar way to concanavalin A (Con A), a well-known lectin from* Canavalia ensiformis* seeds [8]. This preparation has shown interesting biological activities such as immunomodulatory, antitumoral, antiparasitic, and healing agent [9]. Biotechnological applications of Cramoll also involve the characterization of human malignant tissues, affinity matrix for protein purification, and the development of sensors for microbial detection [9]. Cramoll 1 (major isolectin in this preparation) consists of 236 residues with 82% identity with Con A. Cramoll 1 tertiary structure was determined by X-ray crystallography at 1.77 Å and revealed three *β*-sheets connected by loops, known as the jellyroll domain (this topological architecture is essentially identical to Con A) [9]. Recently, the expression of soluble, functional recombinant Cramoll 1 in* Escherichia coli* was reported by our group: rCramoll, which shares the molecular mass, charge density, sugar recognition, and secondary and tertiary structures of pCramoll [10, 11].

In this study the cytoprotective effects of pCramoll and rCramoll against H_2_O_2_-induced oxidative damage in Vero cells were investigated. We found that the cytoprotective effects of these lectins are mediated by the decrease of superoxide species production that prevent the mitochondrial and lysosomal dysfunctions and the DNA damage.

## 2. Materials and Methods

### 2.1. Lectins Purification

pCramoll was purified from seeds of* C. mollis* using Sephadex G-75 column as previously reported [9]. The* E. coli* Rosetta (DE3) was used for the expression of rCramoll using expression vector pET-28a-Cramoll 1 and affinity chromatography (Sephadex G-75 column) [10, 11].

### 2.2. Cell Culture

The monkey kidney fibroblast line (Vero) was maintained at 37°C in an incubator with humidified atmosphere of 5% CO_2_. Cells were cultured in RPMI medium containing 10% heat-inactivated fetal calf serum, penicillin, and streptomycin (100 U/mL), all from Sigma-Aldrich.

### 2.3. MTT Assay

Cell viability was evaluated using the MTT assay, which measures the metabolic conversion of the 3-(4,5-dimethylthiazol-2-yl)-2,5-diphenyltetrazolium bromide (MTT; Sigma-Aldrich) salt to the colored formazan dye. Vero cells (1 × 10^5^/mL) were incubated in a 96-well plate in quadruplicate for 24 h (37°C and 5% CO_2_), treated with lectins (0.625–10 *μ*M) for 30 min and subsequently with H_2_O_2_ (1 mM) for 24 h. At the end of the incubation time, the medium was removed and a MTT solution (5 mg/mL in RPMI) was added to the culture and the cells were incubated for additional 3 h (37°C and 5% CO_2_). Afterwards, the medium was removed again and the intracellular formed formazan product was dissolved in DMSO. The optical density (OD) was measured at 595 nm in a microplate reader (Benchmark Plus, Bio-Rad, California, EUA). Cell viability (CV) was calculated in comparison to the OD obtained by control cell, considered as 100%. The protective effect was calculated following this formula:

(1) $$\begin{array}{c} Protective\;effect\;\left(\%\right)=\frac{\left({CV}_{s}-{CV}_{H_{2}O_{2}}\right)}{{CV}_{H_{2}O_{2}}}*100, \end{array}$$

where CV_s_ is the viability of cells treated with each lectin in the presence of H_2_O_2_; CV_H_2_O_2__ is the viability of cells treated only with H_2_O_2_.

### 2.4. Viability/Cytotoxicity Assay

To confirm the cytoprotective effect of the lectins the Live/Dead Viability/Cytotoxicity kit for mammalian cells (Molecular Probes) was used, following manufacturer's instructions. Briefly, the Vero cells (1 × 10^5^/mL, cultured on a 24-well plate for 24 h at 37°C and 5% CO_2_) were pretreated with both lectins (10 *μ*M, for 30 min) followed by the addition of H_2_O_2_ (100 *μ*L at 1 mM), as inductor of oxidative stress. After 24 h of incubation, the cells were trypsinized, centrifuged at 3000 g for 5 min, washed with PBS and resuspended in 500 *μ*L of PBS containing 2 *μ*L of calcein AM (50 *μ*M) and 4 *μ*L of ethidium homodimer, and then incubated for 20 minutes at room temperature, protected from light. Afterward, the samples were immediately analyzed using FACSCalibur-BD flow cytometer (Becton Dickinson Co., San Jose, CA). For each sample, 10.000 events were collected and the results were analyzed by using the software CELLQuestPro (Becton Dickinson Co., San Jose, CA).

### 2.5. Mitochondrial Superoxide Production

The production of superoxide anion by the mitochondria was measured using the MitoSOX Red mitochondrial superoxide indicator (Molecular Probes). The cells were pretreated with lectins (10 *μ*M, for 30 min) and treated with H_2_O_2_ for 30 min. After the trypsinization and washing, the MitoSOX reagent (1 mL/5 *μ*M) was added and the samples were incubated for 10 minutes at 37°C, protected from light. The cells were washed with warm buffer (three times) and analyzed by flow cytometry (FACSCalibur-BD, San Jose, CA) using the software CELLQuestPro (Becton Dickinson Co., San Jose, CA) for acquisition and analysis of data.

### 2.6. Lysosomal Membrane Stability

An experiment using the lysosomotropic base acridine orange was carried out to measure severe or late lysosomal membrane stability by assessing change in red fluorescence (AO uptake method). The cells were prepared as described above (2.5) and incubated at 37°C in the presence of 1 mM acridine orange (Sigma-Aldrich) and washed in PBS (2X) after 30 min. The pellet containing the cells was resuspended in PBS and analyzed by flow cytometry (FACSCalibur-BD, San Jose, CA; FL3 channel) using the software CELLQuestPro (BD Bioscience, San Jose, CA) for acquisition and analysis of data.

### 2.7. Determination of Mitochondrial Membrane Potential (ΔΨm)

The uptake and retention of the cationic fluorescent dye rhodamine123 was used to evaluate the mitochondrial membrane potential (ΔΨm). Treated cells were trypsinized, washed twice with PBS, and centrifuged at 300 ×g for 10 min. The cell pellet was then resuspended in 1 mL of fresh medium containing 2 *μ*M rhodamine123 and incubated at 37°C in a thermostatic bath for 20 min with gentle shaking. The stained Vero cells were washed and then resuspended in 1 mL of PBS. The variation index (VI) was calculated following this formula:

(2) $$\begin{array}{c} Variation\;Index\;\left(VI\right)=\frac{{FI}_{c}-{FI}_{s}}{{FI}_{c}}, \end{array}$$

where FI_c_ is the mean of fluorescent intensity of control and FI_s_ the mean of treated cells.

### 2.8. CFSE Proliferation Assay

To evaluate the cytoprotective effect of Cramoll on Vero cells the CFSE (carboxyfluorescein diacetate succinimidyl ester) proliferation assay was used. The Vero cells (1 × 10^5^/mL) were stained with CFSE (2.5 *μ*M) and incubated at 37°C for 10 min. The reaction was stopped by the addition of cold RPMI. The cells were centrifuged (300 g for 10 min) and the cell pellet was washed with PBS in the same conditions. Afterwards, the cells were resuspended in RPMI and pretreated with lectins, following the addition of H_2_O_2_ as described (Section 2.5). The proliferation indexes were determined after 72 h by flow cytometry (FACSCalibur-BD, San Jose, CA) using the software CELLQuestPro (BD Bioscience, San Jose, CA) for acquisition and the FlowJo software (Tree Star, Ashland OR) for analysis of data. The proliferation index of control cells was considered as 100%.

### 2.9. Terminal Deoxynucleotidyl Transferase dUTP Nick End Labeling (TUNEL) Click-iT Assay

TUNEL assay was performed using the Click-iT TUNEL Alexa Fluor 488 (Invitrogen) following the manufacturer's protocol. Briefly, the cells were fixed with 4% paraformaldehyde for 15 min and permeabilized (0.25% Triton X-100 in PBS) for 20 minutes at room temperature. Then they were washed twice with deionized water. The TdT reaction cocktail was added and incubated for 1 h, followed by 30 min incubation with the Click-iT reaction solution. The stained Vero cells were washed and resuspended and the fluorescence intensity was analyzed by flow cytometry (FACSCalibur-BD, San Jose, CA) using the software CELLQuestPro (BD Bioscience, San Jose, CA) for acquisition and analysis of data.

### 2.10. Spectroscopic Measurements

The effects of H_2_O_2_ treatment on lectin tertiary structure were evaluated by intrinsic fluorescence using a Jasco FP-6300 spectrofluorometer (Jasco, Tokyo, Japan). Both lectins (10 *μ*M) were incubated with H_2_O_2_ (1 mM). The fluorescence emission of tryptophan was measured at 25°C in a rectangular quartz cuvette with a 1 cm path length, the excitation was at 295 nm, and emission was recorded from 305 to 450 nm using 5 nm band pass filters (for both).

### 2.11. Statistical Analysis

Data is analyzed by one-way analysis of variance (ANOVA) and Turkey test to determine the statistical significance. A *P* value of <0.05 was considered to be statistically significant.

## 3. Results

### 3.1. pCramoll and rCramoll Attenuated the H_2_O_2_-Induced Cytotoxicity

**Figure 1:**
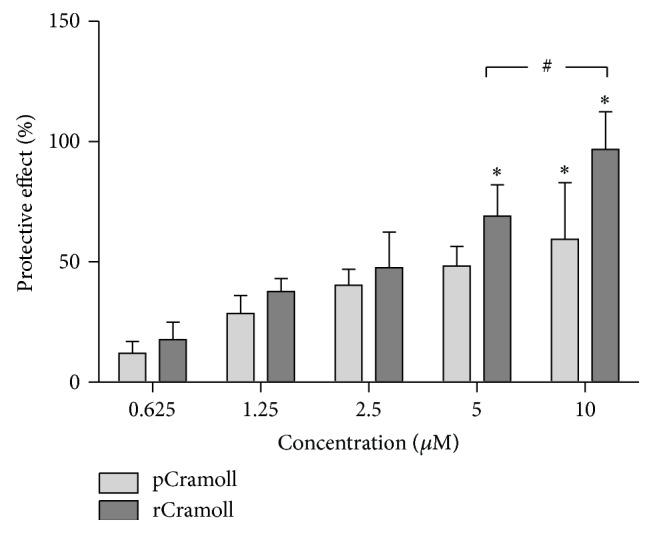
Cytoprotective effects of pCramoll and rCramoll determined by MTT assay. (*∗*) Significant differences between other concentrations. (#) Significant differences between lectins.

The cytoprotective effects of lectins against H_2_O_2_-induced cell death were evaluated by MTT assay, which measures the loss of metabolic activity of cells and it is an early indicator for cell death. Both lectins inhibited the cytotoxicity in a concentration-dependent way (Figure 1). pCramoll showed maximum protective effects at 5 *μ*M (48.36 ± 8.12%) and 10 *μ*M (59.48 ± 23.44%), with no statistically significant differences (*P* > 0.05) between these concentrations. rCramoll induced higher/maximum protection at 10 *μ*M (96.85 ± 15.59%) (*P* < 0.05). It is important to note that rCramoll was more effective than the pCramoll at 5 and 10 *μ*M (*P* < 0.05). Because the best activity of both lectins was achieved at 10 *μ*M we chose this concentration to further investigate the subcellular effects involved in the Cramoll-mediated cytoprotective effects.

**Figure 2:**
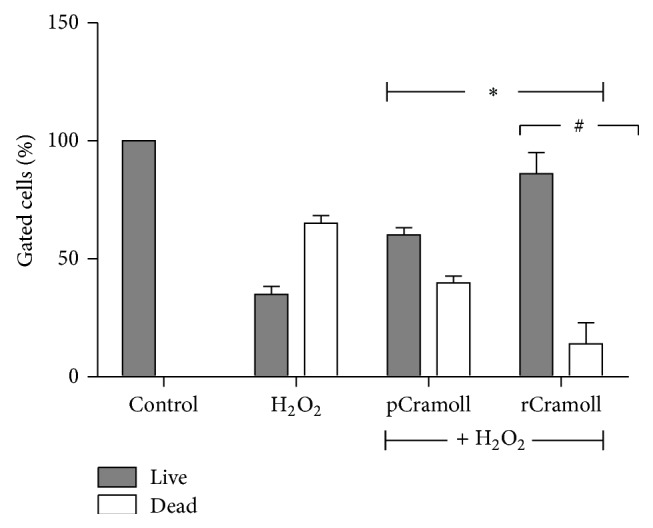
Cytoprotective effects of pCramoll and rCramoll determined by Live/Dead kit using flow cytometry. (*∗*) Significant differences in relation to H_2_O_2_. (#) Significant differences between lectins.

The cytoprotective action of both lectins was confirmed by Live/Dead assay, a flow cytometry analysis that uses the hydrolysis of calcein AM by intracellular esterases of live cells and the ethidium heterodimer (EthD-1) that enter nucleus of cells with damage membranes to discriminate viable from unviable cell, respectively.  Figure 2 shows an increase of cell death when Vero cells were treated with H_2_O_2_ alone (65.04 ± 3.29%) compared to control cells. On the other hand, both lectins reduced the rates of cell death to 39.77 ± 2.93% for pCramoll and 13.90 ± 9.01% for rCramoll.

### 3.2. pCramoll and rCramoll Inhibited the Deleterious Effects of H_2_O_2_ on Cell Proliferation

**Figure 3:**
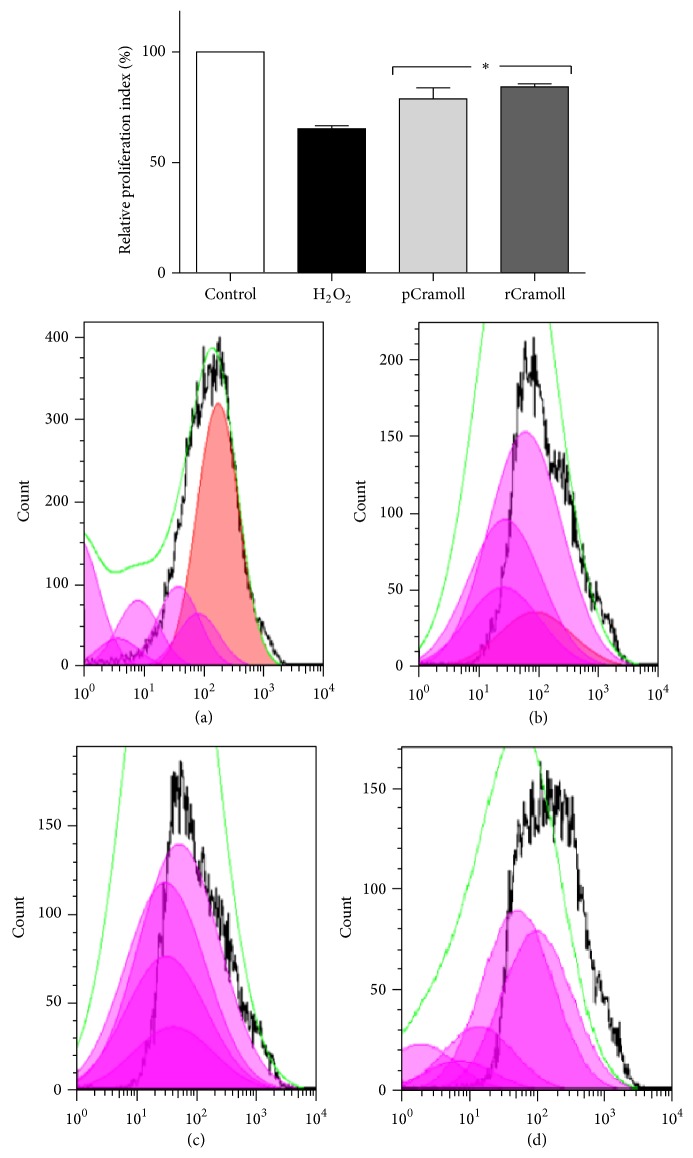
Effects of pCramoll and rCramoll on the deleterious effects of H_2_O_2_ on cell proliferation using CFSE probe, analysed by FlowJo software. (a) Control cells without H_2_O_2_ or lectin treatment. (b) Cells treated with H_2_O_2_. (c) Cells pretreated with pCramoll for 30 minutes prior to H_2_O_2_ addition. (d) Cells pretreated with rCramoll for 30 minutes prior to H_2_O_2_ addition. (*∗*) Significant differences in relation to H_2_O_2_.

To evaluate the cell proliferation a FACS assay using CFSE staining followed by FlowJo analysis was performed. CFSE proliferation assay is based on the ability of CFSE probes to bind to lipids within the cell membrane. After each mitosis, the fluorescence intensity decreases to approximately the half. The H_2_O_2_-treated cells showed, after 72 h, a decrease of proliferation index of 34.71% compared to the control cells. The lectins were able to enhance the proliferation index in the presence of H_2_O_2_ (78.84 ± 4.04% for pCramoll and 84.20 ± 1.16% for rCramoll) (Figure 3).

### 3.3. pCramoll and rCramoll Blocked the H_2_O_2_-Induced Mitochondrial ROS Generation

**Figure 4:**
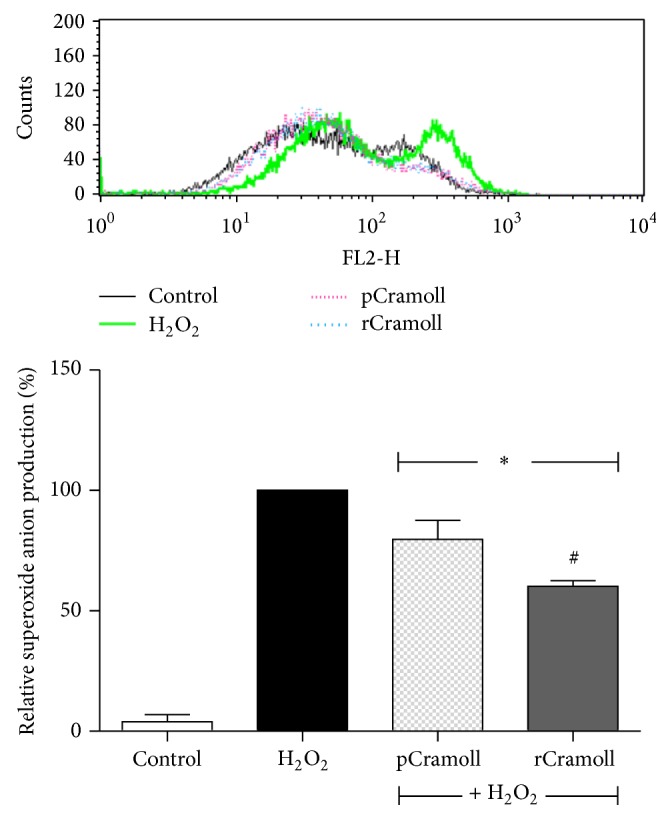
Effect of pCramoll and rCramoll on the H_2_O_2_-induced accumulation of mitochondrial superoxide anion in Vero cells, as observed by flow cytometry. (*∗*) Significant differences in relation to H_2_O_2_. (#) Significant differences between lectins.

As shown in Figure 4, after H_2_O_2_ exposition ROS generation increased more than 52-fold as compared to control. Pretreatment of the cells with 10 *μ*M of lectins for 30 minutes induced significantly the attenuation of mitochondrial ROS production. The reduction rates (in relation to H_2_O_2_) were 20.31 ± 7.82% and 39.84 ± 2.36%, for pCramoll and rCramoll, respectively (*P* < 0.05).

### 3.4. pCramoll and rCramoll Restore the Mitochondrial Membrane Potential (ΔΨm)

**Figure 5:**
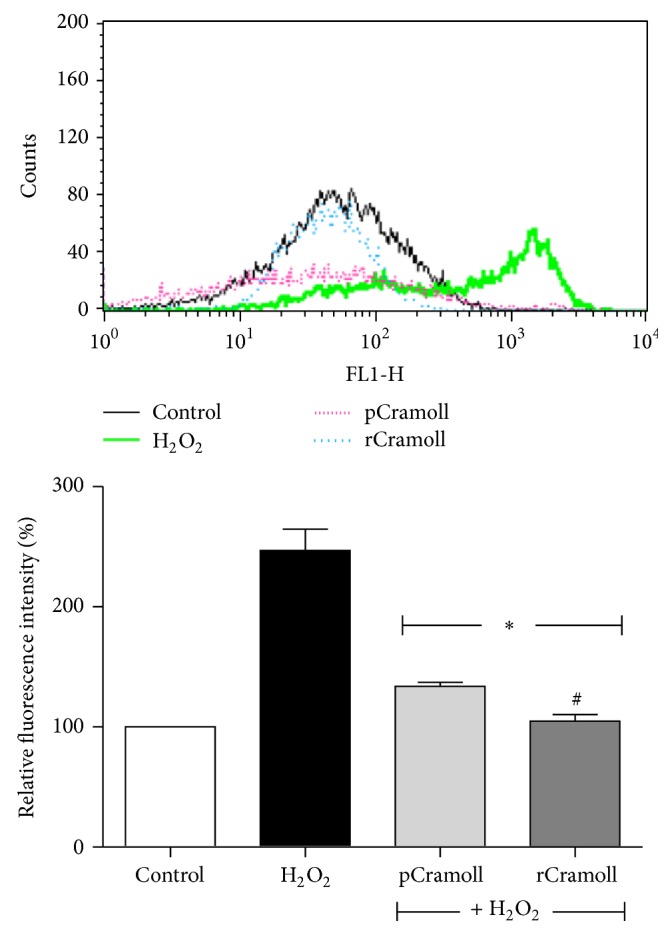
Effect of pCramoll and rCramoll on H_2_O_2_-induced loss of ΔΨm in Vero Cells. (*∗*) Significant differences in relation to H_2_O_2_. (#) Significant differences between lectins.

The alterations in mitochondrial functions were evaluated by the ΔΨm variation index (VI) using rhodamine123. The H_2_O_2_-treated cell exhibited a loss of ΔΨm in relation to the control cells (depolarization, VI: −1.47 ± 0.18). The lectin pretreatments were able to restore the loss of ΔΨm induced by H_2_O_2_ treatment, inhibiting the depolarization (pCramoll, VI: −0.34 ± 0.03; and rCramoll, VI: −0.05 ± 0.06) (*P* < 0.05) (Figure 5).

### 3.5. pCramoll and rCramoll Protect the Lysosomal Damage Induced by H_2_O_2_

**Figure 6:**
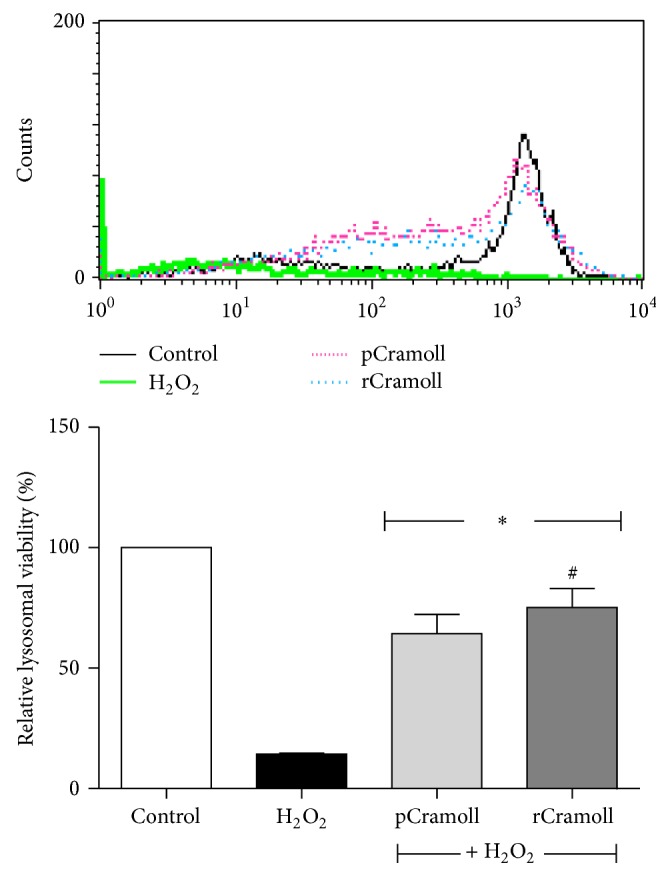
Effect of pCramoll and rCramoll on the lysosomal damage induced by H_2_O_2_ in Vero cells. (*∗*) Significant differences in relation to H_2_O_2_. (#) Significant differences between lectins.

In order to evaluate the lysosomal function the acridine orange (AO) fluorescence was analyzed by FACS analysis. The control cells showed a strong AO fluorescence emission in the red channel, confirming that these cells had intact lysosomes, while H_2_O_2_ reduced in 85.66 ± 0.3% the fluorescence signal. The lectins showed great potential to inhibit the lysosomal membrane permeabilization induced by H_2_O_2_, showing the reestablishment of AO fluorescence signal in treated cells of 64.37 ± 7.98% (pCramoll) and 75.17 ± 7.91% (rCramoll) in relation to the control (*P* < 0.05) (Figure 6).

### 3.6. pCramoll and rCramoll Prevent the Fragmentation of Nuclear DNA

**Figure 7:**
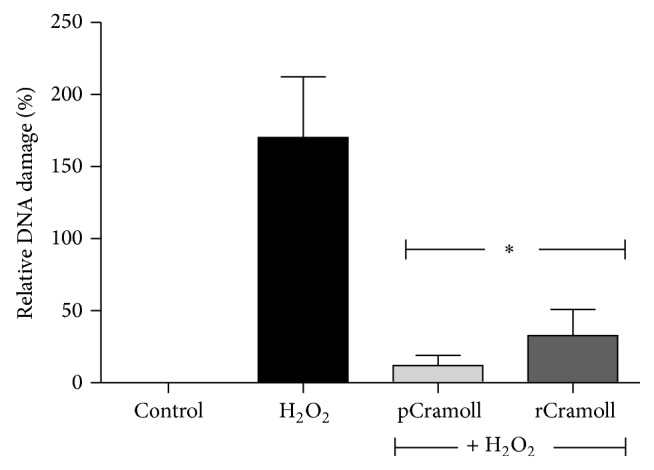
Effect of pCramoll and rCramoll on the DNA damage induced by H_2_O_2_ in Vero cells by TUNEL analysis using flow cytometry. (*∗*) Significant differences in relation to H_2_O_2_.

The formation of DNA ladders is a consequence of a specific nucleosomal-sized fragmentation and is a conventional event in apoptotic process. The TUNEL assay was carried out to detect the extension of DNA degradation in apoptotic cells. DNA damage was quantified in relation to control cells. As expected, no significant DNA injury could be detected in the control cells. On the other hand, H_2_O_2_-treated cells presented an increase in the TUNEL fluorescence intensity, indicative of intense DNA fragmentation (this fluorescence was 170.41% higher than control cell). Pretreatment with both lectins leads to decrease in TUNEL staining in about 2-fold (61.62 ± 1.44% for pCramoll and 55.53 ± 5.86% for rCramoll) (Figure 7).

### 3.7. H_2_O_2_ Decreased Tryptophan Fluorescence Emission of pCramoll and rCramoll but Did Not Alter Their Hemagglutination Ability

Conformal stability of pCramoll and rCramoll (10 *μ*M) after H_2_O_2_ treatment (1 mM) for 24 hours was investigated. Both proteins showed the same behavior: the intrinsic fluorescence emission decreased after exposition to H_2_O_2_ without changing the *λ*_max_, at approximately 330 nm for hydrophobic residues. However, the hemagglutination ability was not modified (data not shown).

## 4. Discussion

In this work we reported the protective effects of native and recombinant Cramoll against H_2_O_2_-induced cell death. The induction of cell death by H_2_O_2_ has been already reported in the literature using different cell types [5, 12–14]. The severe damage caused by H_2_O_2_ is related to its capacity to cross the cellular membranes and react with intracellular metal ions, yielding highly toxic hydroxyl radicals, which are able to cause serious damage to macromolecules, including DNA, proteins, and lipids, and ultimately lead to the cell death [2]. In fact, several diseases had the oxidative stress as the main trigger (cancer, neurodegenerative, and cardiovascular disorders) [1, 15]. In the present work, H_2_O_2_ induced significant toxicity, reducing the cell viability to 34.96% (Live/Dead kit) and suppressing the cell proliferation. The pretreatment with different concentrations of both rCramoll and pCramoll lectins had a significant cytoprotective effect reestablishing the cell viability and proliferation to the rates near to those found in the control cells.

The secondary production of other mitochondrial ROS (such as superoxide) induced by H_2_O_2_ is one of potential mechanisms of cell damage [16]. In this study, we measured the superoxide generation using MitoSOX probe. The treatment of cells with H_2_O_2_ increased significantly the superoxide production by the mitochondria (*P* < 0.05). This effect was attenuated in both lectins-pretreated groups. These results indicate that the anticytotoxic effects of both lectins were related to the inhibition of mitochondrial ROS production.

Mitochondria play a critical role in maintaining the physiology of the cell and its dysfunction is an important pathway in the cell death [17]. It is well known that several proapoptotic mediators (cytochrome c, AIF, Smac/DIABLO, and endoG) are located in the mitochondria. These factors are released into the cytosol where they activate diverse enzymatic reactions that lead to the specific degradation of proteins and DNA during apoptosis [18]. The conservation of ΔΨm is essential for mitochondrial integrity and functions [19]. H_2_O_2_ induces mitochondrial dysfunction by the loss of ΔΨm [20]. Our results showed that native and recombinant Cramoll are able to inhibit the deleterious effects of H_2_O_2_ on the ΔΨm.

Although studies on oxidative-stress-induced cell dysfunction are focused on the alterations in the mitochondrial bioenergetic and proapoptotic compounds release, lysosomes are also susceptible to the oxidative stress and have been associated with necrotic, autophagic, and apoptotic cell death [21, 22]. Different kinds of hydrolytic enzymes (proteases, lipases, nucleases, glycosidases, phospholipases, phosphatases, and sulfatases) are present in this organelle and damage in this structure caused by toxic agents as H_2_O_2_ can lead to the leakage of these enzymes in the cytoplasm resulting in the vanishing of the AO red labeling, as observed in our study [23–25]. The lysosomal proteases such as cathepsins family (A, B, D, and L) are potent activators of apoptotic effectors [26]. The lectins tested in this study were able to prevent lysosomal damage induced by H_2_O_2_ and promoting the cell survival.

Furthermore, the activation of endonucleases leading to the genomic DNA fragmentation is one of the most representative events during apoptosis [27]. The protective actions of the tested lectins against DNA ladder fragmentation induced by H_2_O_2_ in Vero cells were confirmed by TUNEL analysis.

pCramoll and rCramoll share several biophysical properties, the ability to recognize Glc/Man moieties, and the same pH dependent dimer-tetramer equilibrium. However, the tetramers of rCramoll are composed of intact monomers due to the absence of natural fragmentation process, and this characteristic is related to the little enhancement in its stability when probed with acidification, high temperatures, or hydrostatic pressure [10, 11]. As different degrees of protective effects were observed in pCramoll and rCramoll (these differences could be also observed in other biological activities of these proteins [10, 28]), we decided to examine the effects of H_2_O_2_ on their structures using intrinsic fluorescence emission. Cramoll has four tryptophan residues and two of them (40 and 109) are located in the protein core [9]. Tryptophan is easily oxidized by hydrogen peroxide by photooxidation in the presence of oxygen [29, 30]. Oxidative damage distorts the hydrophobic surface and the hydrophobic core of proteins, and partial aromatic amino acid substitution, caused by hydrogen abstraction, results in a decrease in the fluorescence emission. This work showed a decrease in the intrinsic fluorescence emission for both proteins when treated with H_2_O_2_ at 1 mM, suggesting that the different effects of each lectin could be related to other factors, such as sugar affinity.

**Figure 8:**
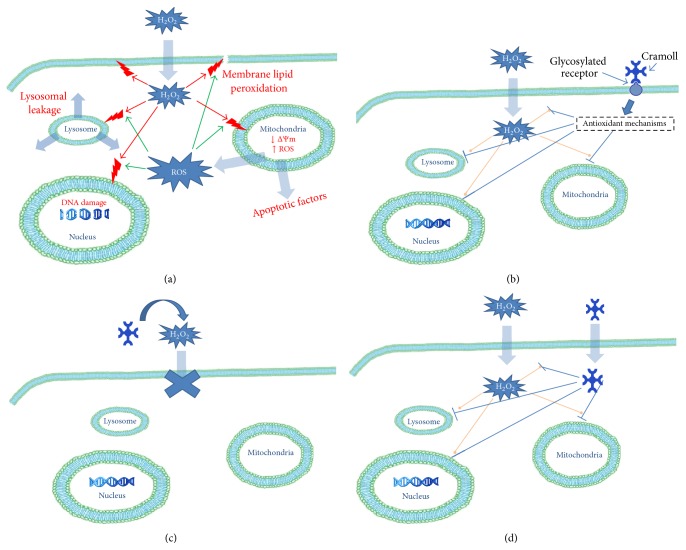
Schematic overview of the protective effects of pCramoll and rCramoll on the H_2_O_2_-induced cell death. (a) H_2_O_2_ can induce cell dysfunction due its capacity to interact directly or indirectly with organelles and cell membrane causing lipid peroxidation, leakage of lysosomal content, DNA damage, decrease in the mitochondrial membrane potential, and increase of mitochondrial ROS production, thereby disrupting cellular function and integrity. (b) The binding of both pCramoll and rCramoll to specific glycosylated targets on the surface of Vero cells or intracellularly can trigger the antioxidant mechanisms of cells that prevent the deleterious effects of H_2_O_2_ on organelles such as mitochondria, lysosomes, and nucleus, promoting cell survival and proliferation. (c and d) Alternatively, both lectins can act directly as H_2_O_2_ or/and secondary ROS scavengers, neutralizing the harmful effects of oxidative stress.

In summary, pCramoll and rCramoll ameliorate the H_2_O_2_-induced oxidative stress and cell death. The protective effects were related to the inhibition of mitochondrial superoxide generation, the reestablishment of ΔΨm, and the blocking of the deleterious effects of oxidative stress on lysosomal integrity and DNA fragmentation, promoting cell survival and proliferation. Although the molecular mechanisms responsible for the antioxidant properties of pCramoll and rCramoll remain to be elucidated, some studies carried out by our and other research groups have demonstrated that some lectins act directly as scavengers of ROS [31–33]. However, we cannot rule out the possibility that pCramoll and rCramoll act indirectly on the antioxidant enzymes triggering detoxification mechanisms that protect the cells against oxidative stress induced by H_2_O_2_ treatment. Further studies are needed to investigate in more detail the molecular mechanisms involved in the protective role of lectins against the damage caused by the oxidative stress (Figure 8).

## Abbreviations

AO: Acridine orange
CFSE: Carboxyfluorescein diacetate succinimidyl ester
Con A: Concanavalin A, lectin from* Canavalia ensiformis* seeds
Cramoll 1,4: Preparation containing isolectins 1 and 4 from* Cratylia mollis* seeds, pCramoll
Cramoll 1: Isolectin 1 from* Cratylia mollis* seeds
DMSO: Dimethyl sulfoxide
H_2_O_2_: Hydrogen peroxide
MTT: 3-(4,5-Dimethylthiazol-2-yl)-2,5-diphenyltetrazolium bromide
O_2_^−^: Superoxide anions
OH^•^: Hydroxyl radicals
PBS: Phosphate buffered saline
rCramoll: Recombinant Cramoll 1
ROS: Reactive oxygen species
TUNEL: Terminal deoxynucleotidyl transferase dUTP nick end labeling
ΔΨm: Mitochondrial membrane potential.
